# 780. A Quality Improvement Initiative to Identify and Close Gaps in Rapid Initiation of Antiretroviral Therapy (ART)

**DOI:** 10.1093/ofid/ofad500.841

**Published:** 2023-11-27

**Authors:** Jonathan Colasanti, Aadia I Rana, J E W E L SAWYER, Melissa Rodriguez, Jenniffer A Meza Jimenez, Jeffrey D Carter, Abigail K Corona, Laura Simone, Leah Molloy

**Affiliations:** EMORY UNIVERSITY SCHOOL OF MEDICINE, Atlanta, Georgia; UAB Heersink School of Medicine, Birmingham, Alabama; Avita Care Atlanta, Atlanta, Georgia; PRIME Education, Fort Lauderdale, Florida; PRIME Education, Fort Lauderdale, Florida; PRIME Education, LLC, Fort Lauderdale, Florida; PRIME Education, LLC, Fort Lauderdale, Florida; PRIME Education, LLC, Fort Lauderdale, Florida; PRIME Education, LLC, Fort Lauderdale, Florida

## Abstract

**Background:**

Treatment guidelines recommend prompt ART initiation after HIV diagnosis, but implementation barriers persist. This project aimed to identify gaps and improve clinical practices surrounding rapid ART initiation in the US.

**Methods:**

Baseline surveys were completed by 130 healthcare professionals (HCPs) in 8 HIV clinics assessing knowledge and practice around ART initiation. Responses informed the development of interactive, live virtual audit-feedback sessions at 8 clinics from 5/2022 – 8/2022. Pre and post-program surveys were administered, team-based action plans developed, and HCPs completed 60- and 120-day follow-up surveys.

**Results:**

Top reported challenges impeding rapid ART initiation were medication adherence for patients with unstable housing/substance use disorders (SUD) (63%), incomplete lab results (33%) and co-infections/opportunistic infections (28%). Knowledge was assessed using case vignettes. For a patient with hepatitis C and SUD, 45% of learners correctly chose to promptly start ART, which improved to 94% after the sessions (p< .001). More HCPs were confident initiating ART for patients with unstable housing/SUD (70%) and before confirmatory HIV testing (74%) after the sessions than before (38% and 39%, respectively, p< .001 each).

Follow-up surveys noted a 10% increase in proportion prescribing ART within 7 days of a positive screening test (32% to 42%) along with increased ART prescribing the same day as a positive test and before additional test results, Figure 1. Action plans implemented after the sessions included coordination with off-site labs, new intake forms, and staff training. HCPs self-reported improvements including appropriate prompt ART initiation (81% and 88%), integrating new patients to avoid loss to follow-up (85% and 88%), and alignment with guidelines (88% and 91%). HCPs identified care coordination (31%) and identifying candidates for rapid ART initiation (31%) as areas of need to improve rapid ART initiation. Challenges reported included scheduling conflicts, timely lab results and coordination with case management.

**Figure 1.**
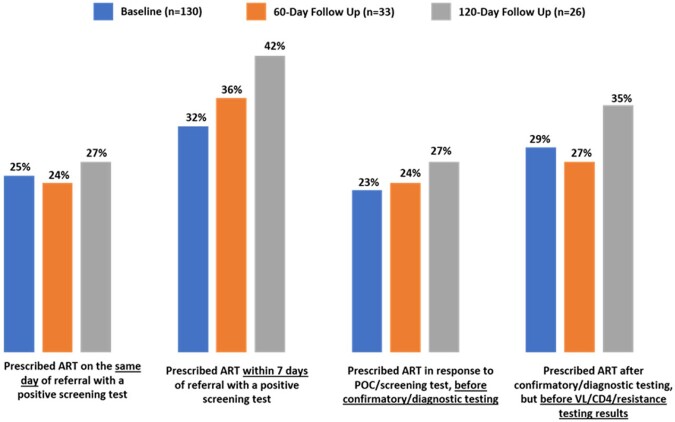
Proportion of HCPs reporting ART initiation practices occurring for >75% of their patients

**Conclusion:**

Tailored implementation discussion with live audit-feedback and action planning led to measurable practice improvements. These outcomes may inform other practice-changing implementation needs.

**Disclosures:**

**Jonathan Colasanti, MD, MSPH**, DKB MED LLC: Honoraria|Prime Education LLC: Advisor/Consultant **Aadia I. Rana, MD**, Merck: Grant/Research Support **JEWEL SAWYER, PA-C, MSHS, AAHIVS**, Janssen: Speakers Bureau|Prime Education: Speakers Bureau

